# The importance of developing palliative care quality indicators for the prison setting: why now, and next steps

**DOI:** 10.1186/s12904-023-01150-3

**Published:** 2023-06-08

**Authors:** Isabelle Schaefer, Nicole Heneka, Michelle DiGiacomo, Stacey Panozzo, Jane L. Phillips

**Affiliations:** 1 University of Technology Sydney, Sydney, NSW Australia; 2 University of Southern Queensland, Toowoomba, QLD Australia; 3 University of Melbourne, Melbourne, Victoria Australia; 4 Queensland University of Technology, Brisbane, Australia

**Keywords:** Prison, Palliative Care, Quality indicators, Service evaluation, Quality improvement

## Abstract

Palliative care is increasingly important in the prison setting, but information about the quality and accessibility of this care is extremely limited. Developing and implementing standardised quality indicators will provide transparency, accountability, and a platform for quality improvement at both local and national levels.

## Introduction

As the number of older people in prison continues to increase internationally, providing palliative care to this population is becoming a critical issue [1–5]. Compassionate release should consistently be the first option explored for people in prison with palliative or end of life care needs. However, many barriers prevent this from often occuring, [6–8] necessitating the provision of high quality primary palliative care within prisons. Where palliative care needs exceed the scope of primary palliative care or rapid deterioration occurs, people in prison should have ready access to an appropriate specialist palliative care service, such as within a tertiary hospital. While the basic palliative care needs of many of these people are managed internally by correctional healthcare providers, [9] providing care to those with more complex or escalating palliative care needs is challenging in the prison environment [10].

Despite these mounting pressures, there is no standardised approach to providing palliative care in prisons, and little data about the quality and accessibility of prison-based care for those with palliative care needs [11]. Compared to other healthcare settings, routine monitoring and reporting of prison healthcare using quality indicators is underutilised, [12] making it difficult to evaluate the care that people in prison receive.

Developing agreed standards and quality indicators for palliative care in prisons is an essential step towards ensuring that high-quality palliative care is accessible and equitable. Quality indicators promote transparency and accountability, and aim to improve targeted outcomes [13, 14]. Without quality indicators that explicitly measure and compare healthcare to an agreed benchmark, it is difficult to highlight areas where care does not meet patient needs or comply with accepted standards [13]. Designing these indicators can be informed by lessons learned from similar efforts in other settings within the community.

Examining the limited number of published prison healthcare indicator sets, along with the community-based palliative care indicators will help inform the development of prison-based palliative care indicators.

### Collecting evidence: availability of data

A substantial barrier to the development and implementation of palliative care indicators is the limited availability of standardised data for comparison between jurisdictions in both community palliative care [15, 16] and general prison healthcare [17, 18]. National palliative care-oriented datasets in the community are not uniformly implemented, even in countries rated as providing high quality palliative care; although increasing standardised data collection for service improvement is a commonly reported goal [15]. Even within established systems where palliative care activity data is routinely collected, inconsistent reporting within and across services makes palliative care quality difficult to assess [19, 20].

Internationally, prison health data is highly valued [21–24] but inconsistently collected and not readily accessible, [17] limiting performance measurement within prison healthcare systems [18, 21–23, 25, 26]. Improving data collection items and strategies is an essential step towards the implementation of quality indicators.

The capacity to capture regular, clinically useful data on the structure, processes and outcomes of prison-based palliative care should be improved [21, 27]. Health information technology that includes automated extraction of data from electronic health records is an efficient solution to costly and time-consuming manual data retrieval, and provides flexibility in the frequency and focus of data collection. However, these features are often unavailable or underutilised in the prison system [21, 22, 27]. Investment in health information technology infrastructure will expand data collection capacity across jurisdictions and enable more comprehensive reporting at regional and national levels.

While such changes are in progress, initial development of indicators should take a pragmatic approach to account for limited resources and health information systems. Indicators that use easily extractable data from current systems, are representative of palliative care in the context of the prison environment, and already recognised as a valuable measure of health in community settings should be prioritised [17, 27–29]. This will foster the gradual and sustainable development of data collection, extraction, analysis and feedback systems for palliative care quality improvement.

### Finding the balance: types of indicators

A further important consideration is the type of indicators to be developed. Currently, both community palliative care and prison indicator sets show an unequal distribution of indicators that gauge the structure of the healthcare system, process of care and care activities and outcomes of patients who receive care [13]. Community palliative care indicators tended to focus on processes and outcomes of palliative care rather than structure measures; which have increased over time [15, 30, 31]. Prison-based indicators heavily favoured process measures that described delivery of care [27]. Whilst it is recognised that an indicator set does not require equal numbers of each type, each indicator type contributes different and important information about healthcare, and using some combination of the three balances the strengths and weaknesses of each [32].

Outcomes indicators are considered the “…ultimate validators of the effectiveness and quality of medical care.” (p694) [32]. Therefore, the growing emphasis of outcomes indicators in community palliative care sets should be better reflected in future prison-based palliative care indicators, given they are largely absent in the prison setting [12]. Though patient-reported outcome and experience measures are arguably more difficult to gauge, more complex to interpret and do not necessarily directly translate to improvement strategies, they provide an important holistic reflection of healthcare quality, rather than a single process within the larger system [33].

Structural measures of quality palliative care may also be of considerable use to understand variabilities in facilities and equipment [33] between correctional facilities, jurisdictions and countries. Lack of staffing and equipment is a common problem, [34–38] but resource and organisation measures are rarely incorporated into current prison quality indicator sets [12]. Setting standards for basic education in palliative care skills for clinicians, ensuring the availability of equipment such as pressure mattresses and accessibility features in the physical environment; and assessing the proportion of prisons that comply with these standards may help to ensure that basic elements of palliative care are available in every prison. In turn, this may reduce the need to transfer people to hospital for palliative care needs that would normally be managed outside of an acute care environment.

### Reflecting environmental differences: indicator development

Differences between community and prison palliative populations demographics and diagnoses, [39–41] and the lack of evidence-based clinical guidelines for the prison setting mean that future development of prison palliative care indicator sets will likely require a combination of adoption of community indicators and consensus-based adaptation or development of prison-specific palliative care indicators. Greater national and international collaboration would minimise duplication of effort in developing prison palliative care indicator sets, as is recommended in the community [15].

However, there are no clear parameters describing how to determine which existing community-based indicators are suitable for the prison environment, which elements are acceptable to adapt without altering the intent or validity of the original indicator, or how to identify instances where development of novel indicators is essential for use in the prison environment. Without established criteria to standardise these choices there is a risk that newly developed sets will drift from the evidence base and community care norms, such that prison-based quality measures no longer reflect best-practice care.

Using prison health evaluation models such as the *WHO Prison Health Framework* [42] or the *Five Nations model for prison health surveillance* [22] that incorporate prison-specific considerations into core principles of community health may help to generate indicator sets that are balanced between evidence-based community indicators and setting-appropriate prison indicators.

In the absence of evidence-based guidelines, broad, iterative consultation with a variety of stakeholders will ensure that indicators developed are feasible, focus on recognised unique needs within prison system and population, and are appropriate for use between different prison systems. Co-designing indicators with key external and internal correctional stakeholders will help focus the development on prison-specific palliative care health issues and ensure that all new indicators reflect evidence-based community standards where possible.

Involvement of people with lived experience of incarceration could also be explored to support inclusion of the patient perspective, as is now an increasing focus in community-based healthcare evaluation. Mechanisms such as ‘citizen’s juries’ comprised of people in prison recently used for health priority-setting in Australian prisons may be a useful tool for incorporating the patient voice [43].

## Conclusion

Standardised, prison-based palliative care indicators will provide valuable data to scope and improve the quality and accessibility of care. A collaborative approach to indicator development will reduce the burden of developing indicators and allow core indicators to be collected and compared between regions or countries, while retaining the flexibility to include indicators specific to local needs. During the initial stages of development, prioritisation is key as practical considerations will limit the number of indicators that can be operationalised. Starting small with simple, clinically useful measures and building on incremental progress will be the most sustainable approach towards a comprehensive indicator set. Taking practical steps to improve palliative care for people in prisons that draw from lessons learned in the community setting will help to slow the growing gap between patient need and available care.

## Data Availability

Not applicable.
