# Correction: The importance of developing palliative care quality indicators for the prison setting: why now, and next steps

**DOI:** 10.1186/s12904-023-01210-8

**Published:** 2023-07-07

**Authors:** Isabelle Schaefer, Nicole Heneka, Michelle DiGiacomo, Stacey Panozzo, Jane L. Phillips

**Affiliations:** 1 University of Technology Sydney, Sydney, NSW Australia; 2 University of Southern Queensland, Toowoomba, QLD Australia; 3 University of Melbourne, Melbourne, Victoria Australia; 4 Queensland University of Technology, Brisbane, Australia

**Correction: BMC Palliat Care 22, 69 (2023)**

**https://doi.org/10.1186/s12904-023-01150-3**

Following the publication of the original article [1], it has been reported that Dr. Jane Phillips should also be affiliated to affiliation 1. Moreover, the following sentences in the Introduction section should be updated

from

However, the reality of the many barriers to release near the end of life [6–8] necessitates the provision of high quality primary palliative care within prisons….

…While the basic palliative care needs of many of these people are managed internally by correctional healthcare providers, [9] providing care to those with complex or escalating palliative care needs is more challenging in the prison environment [10].

into

However, many barriers prevent this from often occuring, [6–8] necessitating the provision of high quality primary palliative care within prisons….

…While the basic palliative care needs of many of these people are managed internally by correctional healthcare providers, [9] providing care to those with more complex or escalating palliative care needs is challenging in the prison environment [10].

The original article has been updated.
